# Diseases Admitted under the Care of the Physicians of the Westminster Hospital, from the 21st of June, to the 23d of July, 1800

**Published:** 1800-08

**Authors:** 


					Difeafes admitted under the Care of the Pbyjicians of the Wejim'mji;r
Hofpital, from the llji of June, to the 2$d of July, 1800.
Continued Fever - - - z6
Quotidian ----- i
Pleurify ------ z
F.ryfipelas - - - - i
Meafles f ----- 3
Chicken Pox - - - - 1
Scarier Fever - - - 1
Amenorrhoqa - - 5
Anafarca 4
Angina Pe?loris - - - - 1
Aftheuia ----- 10
Afthma ------ 3
Catarrh ------ 2
Cephalaea ----- 5
Cholera ------ 3
Colic - -- -- -- 2
Convalfions - - - -l - 2
Cough ------ g
Diarrhoea - - - - - 4
Dyfpepfia - - - - - 3
Dyfpncea ------ 2
Dyfuria ------ I
Epilepfy ------ 2
Gaftrodinia ----- 2
Hzematemefis - - - - 2
Hxmorrhois - I
Hooping Coagh - 3
Hydrothorax - - - ~ 3
Itch ------- 9
Jaundice ----- 1
Lepra Graec ----- 2
Leucoirhcea ----- 3
Menorrhagia - - - - 3
Obftipatis - - - - - z
Paralyfis - - - - - 3
Phthliisj ------ 5
Pleurodynia ----- 1
Podagra ------ z
Rheumaufia
Rheumatifm - - - - 5
Struma ------ *
Syncope ------ I
Syphilis ------ j
Tinea ------ %
Worms - - - - - - 4

				

